# The Position of Women at Cambridge

**Published:** 1920-11-13

**Authors:** 


### THE EDITOR'S LETTER-BOX.

**Correspondence on all subjects is invited, but we cannot in any way be responsible for the opinions expressed by our correspondents, who must give their name and address as a guarantee of good faith, but not necessarily for publication. Correspondents are reminded that brevity of style and conciseness of statement greatly facilitate early insertion.**

### The Position of Women at Cambridge.

*To the Editor of* The Hospital.

Sir,—May I point out that if Cambridge admits women to membership of the University in accordance with the proposed statute, she will have complete control over the numbers of women admitted, in that any further expansion of the present colleges, or the institution of any other women's college or public hostel, must meet with the sanction of the University. The University will also be free to formulate and enforce any chaperonage or other disciplinary rules which may be considered necessary or desirable in connection with the relations between the undergraduates and the women students.

At present Cambridge, far from being a University entirely for men, is a mixed University in all but name. The women are there, but the University has no control over the expansion of their colleges or over their discipline. If women are given formal admission to the University this control is gained. After all, as someone has pointed out, a women with a degree takes up no more room than a woman without one!

It cannot be denied that much of the opposition to the admission of women is derived purely from a spirit of prejudice. One of your correspondents is evidently an unfortunate victim to this spirit. An attitude of mind is no argument.—Yours faithfully,

A Member of the Public.

